# Biogenic Weathering: Solubilization of Iron from Minerals by Epilithic Freshwater Algae and Cyanobacteria

**DOI:** 10.3390/microorganisms6010008

**Published:** 2018-01-15

**Authors:** George E. Mustoe

**Affiliations:** Geology Department, Western Washington University, Bellingham, WA 98225, USA; mustoeg@wwu.edu; Tel.: +1-360-650-3582

**Keywords:** algae, bioassay, biogenic weathering, biotite, chelation, cyanophyte, epilithic, iron minerals

## Abstract

A sandstone outcrop exposed to freshwater seepage supports a diverse assemblage of photosynthetic microbes. Dominant taxa are two cyanophytes (*Oscillatoria* sp., *Rivularia* sp.) and a unicellular green alga (*Palmellococcus* sp.). Less abundant taxa include a filamentous green alga, *Microspora*, and the desmid *Cosmarium*. Biologic activity is evidenced by measured levels of chlorophyll and lipids. Bioassay methods confirm the ability of these microbes to dissolve and metabolize Fe from ferruginous minerals. Chromatographic analysis reveals citric acid as the likely chelating agent; this low molecular weight organic acid is detectable in interstitial fluid in the sandstone, measured as 0.0756 mg/mL. Bioassays using a model organism, *Synechoccus elongates* strain UTEX 650, show that Fe availability varies among different ferruginous minerals. In decreasing order of Fe availability: magnetite > limonite > biotite > siderite > hematite. Biotite was selected for detailed study because it is the most abundant iron-bearing mineral in the sandstone. SEM images support the microbiologic evidence, showing weathering of biotite compared to relatively undamaged grains of other silicate minerals.

## 1. Introduction

Relationships between living organisms and the lithosphere have received considerable attention from geoscientists because organisms are known to have occurred in association with rocks and sediments for more than three billion years of the Earth’s history. The lithosphere provides the basic source of mineral nutrients for all organisms, and some microorganisms have acquired the ability to dissolve minerals. This report describes observations and laboratory investigations of surface-dwelling (epilithic) microbes that are able to dissolve iron from ferruginous minerals, and to metabolize this element. The studies focus on the weathering of biotite, the dominant iron-bearing mineral in the sandstone bedrock at the study site, located in northwest Washington, USA. At this locality, the rock surface is saturated with freshwater seepage from overlying unconsolidated sediment, an environment that provides a habitat for a variety of surface-dwelling microorganisms, resulting in a nearly continuous biofilm.

This study is significant because it demonstrates the ability of epilithic algae to obtain Fe from their mineral substrate; chelating abilities of bacteria, lichen, and fungi are well-documented, but few data have been available for algae. Also, the research demonstrates the value of the bioassay method for studying bioweathering and iron metabolism.

## 2. Project Description

This report describes a sandstone outcrop on the shores of Puget Sound, where fresh water seepage keeps the surface perpetually moist. The rock provides a habitat for a variety of photosynthetic organisms. Laboratory investigations were conducted to determine if these microbes are involved in biogeochemical weathering.

### Site Description

**Figure 1:**
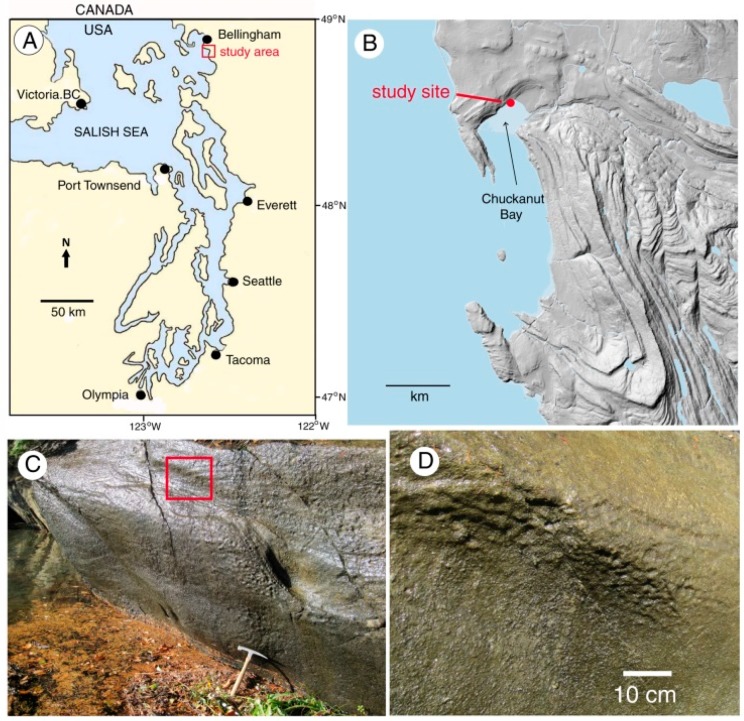
(**A**). Location map; (**B**). Lidar view of Chuckanut Bay and adjacent hill slopes. Topography is strongly related to the highly folded Chuckanut Formation sedimentary rocks; (**C**). Study site outcrop photographed at high tide (2.9 m above mean tide line). Ocean surface is partly obscured by floating plant debris. The rock hammer rests on beach sand; (**D**). Close-up view of outcrop surface (area shown in red outline in (**C**)), showing water saturation and biofilm of endolithic microbes.

**Figure 2:**
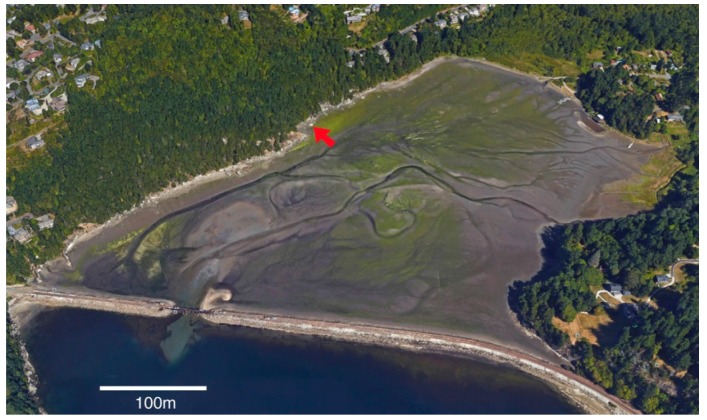
Chuckanut Bay at low tide. Study site is marked with an arrow.

Chuckanut Bay is located in Whatcom County, Washington, near the southern city limits of Bellingham (Figure 1). Bedrock is composed of Eocene arkosic sandstone of the Chuckanut Formation. The outcrop examined for this study lies at the head of the shallow intertidal embayment. Chuckanut Bay is an unusual coastal landform for Puget Sound, being protected from wave action by a railway track bed built in 1901; tidal influx and outflow are limited to a narrow channel (Figure 2). The source of moisture at the study outcrop is fresh water seeping from the adjacent hillside; the surface is inhabited by a freshwater microflora, not marine life forms.

## 3. Previous Work

The ability of biologically produced compounds to dissolve minerals is known from laboratory studies and field observation.

Biogenic activity has been of interest because of the roles microorganisms play during large-scale geologic processes. Kaolin deposits of southern Germany and the Czech Republic have been ascribed to the alteration of granite by organic acids derived from overlying lignite [1], and bauxite deposits of Arkansas, USA have been explained as weathering products of nepheline syenite from organic decomposition related to neighboring lignite beds [2]. High alumina clay in the Negev region in Israel may have resulted from the selective leaching of basalt by dissolved organic matter from soil [3].

Living organisms are an important force during rock weathering. The solubilization of minerals by biologically-derived compounds has been recognized for lichen, fungi, bacteria, cyanobacteria, and degradation products from higher plants. The ability of lichens to secrete compounds that attack minerals is well documented [4,5,6,7,8,9,10,11,12]. Lichens may also cause rock deterioration as a result of expansion and contraction during wetting and drying cycles [13,14,15]. Bacteria are also agents of biogenic weathering [16,17,18,19,20,21,22,23], but fungi are perhaps the most important organism [24,25,26,27,28,29]. Research interest has been stimulated by the realization that fungi and other microbes can be useful for mitigating sites polluted with heavy metals, radionuclides, asbestos, and other hazardous materials [26,30,31,32]. Higher plants can be a cause of biogenic weathering for several reasons. Roots growing along fractures can cause structural damage to bedrock, and rootlets are commonly associated with mycorrhizal fungi that release compounds that solubilize nutrient minerals from adjacent soil [33,34,35]. Perhaps the greatest factor comes after death, when the degradation of plant tissues produces soil organic matter that contains powerful chelating agents. The chemical mechanisms involved in these biogenic weathering processes are described below.

### Chemical Mechanisms

Iron-chelating compounds secreted by organisms are sometimes referred to as siderophores [36,37,38,39]. Although iron is abundant in many natural environments, the element is commonly present as oxides that have a very low solubility, and it is thus unavailable for metabolic use. The secretion of siderophores allows microorganisms to obtain iron from ferric minerals by the formation of soluble Fe^3+^ organometal complexes.

**Figure 3:**
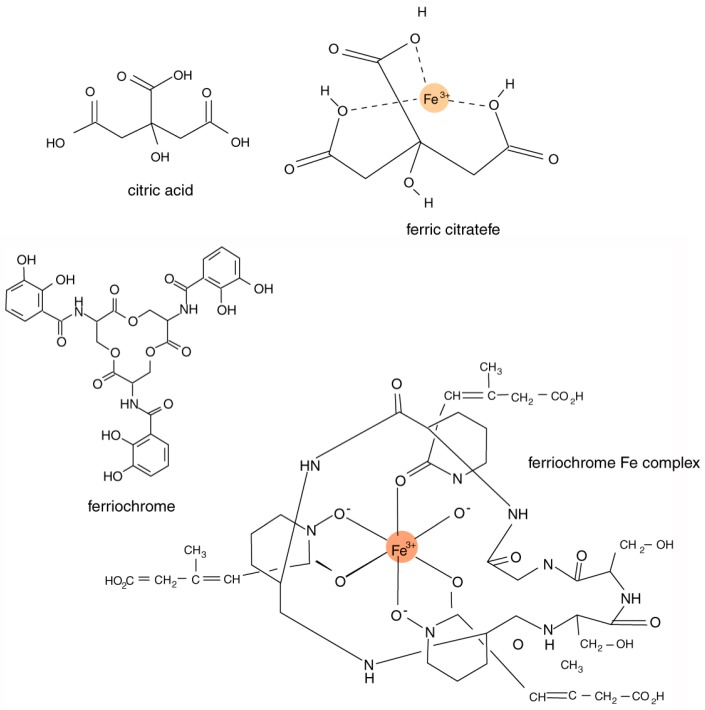
Dissociation of hydroxyl functional groups produces sites that can bond to Fe^3+^ ions. Stereochemistry is an important factor; organometal complexes may have structures very different from simple molecular diagrams. Citric acid is a carboxylate; ferriochrome, a siderophore involved in iron transport for fungi and bacteria, is a hydroxamate. For a given siderephore, Fe complexes generally consist of multiple structural variants that share a basic formula. In solution, these isomers can coexist in equilibrium [44,45].

The chemical composition of siderophores is highly variable, including peptides, chatecolates (phenolates), hydroxamates, and carboxylates. These compounds typically have an ability to form stable octahedral complexes with Fe^3+^ ions. The most important structural characteristic of these complexing agents is their tendency to dissociate in aqueous solution to donate two protons (diprotic acids) or three protons (triprotic acids), allowing the formation of complexes with multivalent metals (Figure 3). Bacteria and fungi produce a diverse variety of catecholate and hydroxamate siderophores [40,41,42]. In algae and higher plants, carboxylic acid siderophores predominate [43].

For organic acids, three principal reaction pathways may be responsible for mineral decomposition: these involve attack by hydronium ions, low molecular weight organic acids (LMWOR), and high molecular weight organic acids (HMWOR).

Hydrogen ions may be involved in biogenic weathering when organic acids are present in concentrations sufficient to produce low pH conditions. However, as noted later, the effectiveness of organic acids for rock weathering primarily comes from their ability to form organometal complexes where multivalent metals form ligands with carboxyl functional groups, not from hydronium ion activity [46]. Hydronium ion attack may occur when carbonic acid is present as a result of the dissociation of biologically-produced CO_2_. This gas is produced during the metabolic process of respiration, but in plants exposed to light, CO_2_ is utilized as a substrate for photosynthesis, along with additional CO_2_ obtained from the surrounding atmosphere. Biochemical processes vary between times of darkness and light, but on net balance, photosynthetic organisms consume CO_2_ and produce O_2_. For this reason, carbonic acid attack on minerals is not likely to be a major force on surfaces inhabited by algae or cyanophytes.

Hydronium ion attack may occur from the release of oxalic acid, derived from calcium oxalate present in the cells of many plants. In addition, oxalic acid may also serve as a chelating agent [45]. Calcium oxalate occurs in the cells of many plants, from microscopic algae to giant gymnosperms, and in cyanophytes and fungi [47,48,49]. Formed from glucose by an enzymatic process, calcium oxalate may accumulate to form considerable quantities of crystals within individual cells, a phenomenon that may provide physical protection against predation [50]. Metabolically, calcium oxalate is a mechanism for regulating calcium levels in tissues and organs [51,52]. Oxalic acid release by lichens and fungi can be important for biogenic weathering, and this compound is known to occur in some microscopic algae [53,54,55]. However, oxalic acid production is not well documented for microscopic algae and cyanophytes; for these microbes, the potential benefits of oxalate production are probably very different compared to larger life forms. For microbes that release organic acids as chelating agents to obtain nutrient elements, the most likely agents are carboxylic acids, as discussed below.

In general, the dominant chemical process for biogenic weathering is chelation, a process where organic molecules react with multi-valent ions on mineral surfaces, solubilizing these elements to produce organometal complexes. The principal chelating agents are organic acids belonging to two groups: high molecular weight (HMWOA) and low molecular weight (LMWOA).

High molecular weight organic acids (HMWOA) are primarily degradation products of dead plant matter, producing humic and fulvic acids that are present in soils, peat, and water. Because they are degradation products, these organic acids are exceptions to a definition of biochemicals as compounds that are produced by living organisms. They are mentioned here because they originate from true biochemical precursors, and because they potentially provide a source of soluble nutrients for living cells.

Humic acids have an aromatic hydrocarbon framework with associated phenolic and carboxylic functional groups that can react with divalent and trivalent metals to produce chelate complexes. Fulvic acids have structural similarities to humic acids, but they are lower in molecular weight, and are more biologically reactive [56]. These compounds are important for the breakdown of minerals in soils [57,58].

Low molecular weight organic acids (LMWOA) include members of two groups: carboxylic acids produced during the citric acid cycle (Krebs cycle), and phenolic acids (lichenic acids). Lichenic acids are phenolic compounds formed as secondary metabolic products.

In this report, the study site is a sandstone outcrop saturated with groundwater. Fresh-water algae inhabit the exposed surface. In this environment, biogenic weathering is most likely caused by the chelating effects of LMWOA generated during the citric acid cycle (Krebs cycle). This reaction sequence is a fundamental process for carbohydrate oxidation for all aerobic organisms. Pyruvate produced from glucose during anaerobic glycolysis is utilized during the aerobic Krebs cycle, where intermediate metabolites are citrate, isocitrate, oxalyosuccinate, ketoglutarate, succinate, fumarate, malate, and oxalyacetate. These tricarboxylic acids all have the potential to be effective chelating agents for multivalent metals because of their multiple carboxyl functional groups.

The release of metabolic intermediates would seem to be detrimental to efficient metabolism; the benefit may be that that the release of LMWOA gives an organism the ability to obtain nutritional elements from nearby mineral surfaces. However, the energetic efficiency of metabolism may be very different for organisms that live directly on bare rock or unweathered soil, compared to organisms that are epiphytic or parasitic, or plants rooted in fertile soils. In the latter instances, mineral nutrients are obtained either from a host, or from organic chelates formed by reactions of minerals with dead organic matter (HMWOA). In contrast, organisms dwelling in barren environments need to obtain essential elements from a primary source, e.g., rock-forming minerals.

The most abundant tricarboxylic cycle acid to be released outside cells is usually citric acid (C_6_H_8_O_7_). Indeed, the commercial production of citric acid typically relies on the fermentation of various sugars by the fungus *Aspergillus niger* [59,60,61]. Experiments involving the effectiveness of fungal culture in mineral dissolution indicate that solubilization can be caused by the production of citric acid by the organisms [24].

For LMWOA, the nature of the chemical reaction varies with the chemical composition of the minerals and the organic acids. In sulfides and oxides, solubilization occurs when metal cations are removed from the lattice. For silicates, the decomposition sequence encountered under inorganic conditions may be quite different from the sequence that occurs in the presence of organic acids. The alteration of biotite provides an example. A common inorganic pathway for the weathering of biotite involves the transformation to vermiculate when hydrated cations cause interlayer expansion. In the presence of organic acids, metal ions in the octahedral layer are removed, leaving behind a fragile matrix of amorphous silica. This relict silicate lattice will disintegrate upon mechanical disturbance of the mineral grain [62].

Studies of dissolution of iron from ferruginous minerals and from granodiorite show that organic acids of biologic origin can dissolve iron from the lattices of certain minerals within minutes [63]. The amount of solubilization bears no direct correlation to the pH of the aqueous solutions, evidence that the dissolution of metal cations is the result of chelation, not the action of hydronium ions produced by the dissociation of these organic acids [46].

Solubilization of minerals in the presence of organic chelating agents may be enhanced relative to solubility under inorganic conditions. Most minerals show small but measurable solubility in pure water, but equilibrium is normally reached before significant lattice damage occurs. However, organic compounds present in solution may react with ions as they are released from the mineral, producing soluble organic complexes. Such reactions would push the equilibrium toward the release of additional ions, so that the mineral could continue to dissolve indefinitely without reaching a saturation point.

The bioavailability of Fe commonly involves chelation effects, but this is not the only mechanism. In aquatic and marine environments, phytoplankton may obtain this micronutrient from unchelated Fe [64,65]. Iron bioavailability may also be facilitated by the reduction effects of organic ligands [66,67]. This is a reminder that although the present study provides data for understanding iron bioavailability for microbes growing on rock surfaces, it is not a model for global Fe uptake.

## 4. Site Geology

This report focuses on the microbial solubilization of iron from biotite because this mineral is the most abundant ferruginous material in Chuckanut Formation arkose, constituting up to 6% by volume of the total rock [68]. Also, the clastic grains are set in a matrix of micaceous material, so the dissolution of biotite is an important step during weathering. The typical composition of the rock is shown in Table 1.

## 5. Microbiology

**Figure 4:**
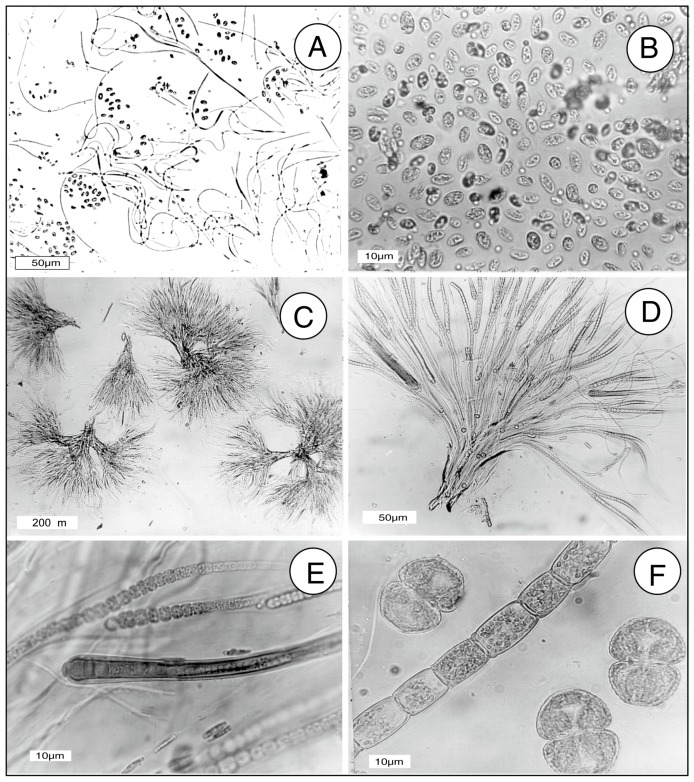
Microorganisms from the rock surface at the Chuckanut Bay outcrop. (**A**), *Oscillatoria* sp. (Cyanophyta) and *Palmellococcus* sp. (*Chlorophyta*); (**B**), *Palmellococcus* sp.; (**C**,**D**), *Rivularia* sp. (Cyanophyta); (**E**), *Rivularia* sp. trichomes; (**F**) filamentous *Microspora* sp. (Chlorophyta), and three desmid cells, *Cosmarium* sp. (Charophyta).

Samples were collected as scrapings from the rock surface, collected in springtime (early May). The most abundant microrganisms (Figure 4) were identified as filamentous cyanophytes *Oscillatoria* sp. and *Rivularia* sp., and unicellular *Palmellococcus* sp. (Chlorophtya). Less abundant taxa included filamantous *Microspora* sp. (Chlorophyta) and the desmid *Cosmarium* sp. Identifications were made based on morphology using the information in [70,71,72,73,74].

### 5.1. Chlorophyll and Total Lipid Analysis

**Figure 5:**
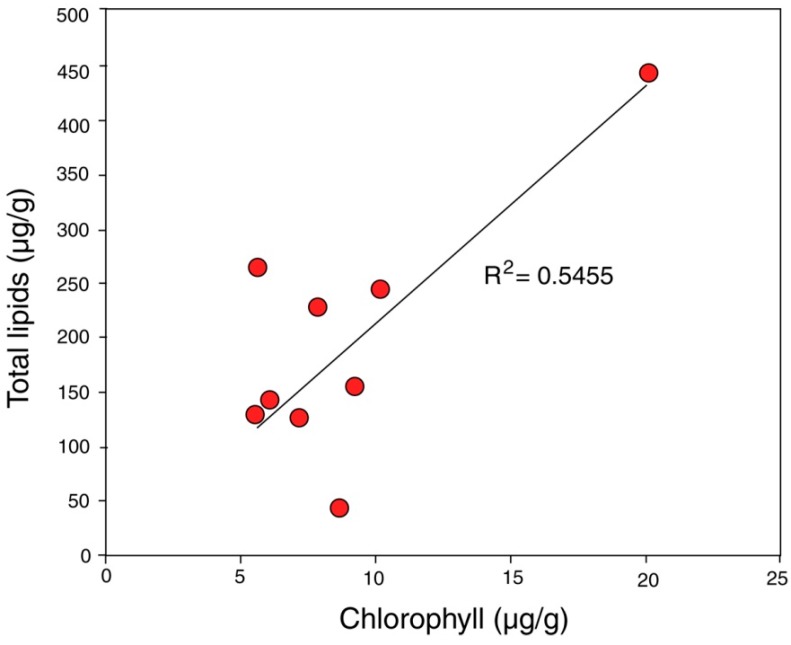
Chlorophyll-lipid ratios show a weak positive correlation. *R*^2^ = correlation coefficient based on the sum of the squares of regression residuals.

Evaluating biologic activity began with the determination of chlorophyll and total lipids (Table 2) in small rock chips collected in a horizontal line across the face of the outcrop using the methods described in Appendix A. Chlorophyll analysis provides a useful method for recognizing the presence of living photosynthetic organisms, because once released from living cells, this pigment degrades rapidly [75]; Total lipids likewise represent biologic activity, galactolipids and phospholipids being important structural components of cell membranes [76]. Because of their greater chemical stability, total lipid values may represent contributions from both living cells and accumulated degradation products. This may explain the weak positive correlation of chlorophyll and total lipids (Figure 5). However, the chlorophyll-lipid ratio may show wide variation among different taxa, because the thicknesses of lipid-rich cell walls may vary. The significance of these data is that they provide numerical evidence for recognizing microbial activity at the rock surface.

### 5.2. Organic Acid Analysis

Thin layer chromatography was used to analyze low LMWOR at the outcrop surface zone. To obtain a sufficient sample size, a total of 80 g of rock chips were collected from a horizontal line across the face of the outcrop in the zone where the rock was visibly covered by epilithic organisms. Citric acid was the only LMWOR detected. Quantitative analysis (Appendix A) revealed a citric acid content of 0.575 mg, representing the total amount of this compound in the moist rock sample. After drying at 110 °C, a duplicate 80 g rock sample was observed to have a weight loss of 7.6 g, indicating the original amount of interstitial fluid. From this information, the original citric acid concentration was calculated to be 75 mg/7.6 mL, equivalent to 0.0756 mg/mL. This value was used in subsequent laboratory experiments.

## 6. Experimental Evidence

Several experiments were conducted to investigate possible biogenic weathering at the study site. Procedure details for all methods used in this study appear in Appendix A. The first experiment measured the dissolution of Fe from biotite exposed to citric acid at the concentration measured from interstitial water. Iron was selected because ferruginous minerals, particularly biotite, are major components of the intergranular cement that lithifies the arkosic sandstone. Also, Fe is an essential nutrient for microbes, because this element is a cofactor for many enzymes. Indeed, low Fe availability is commonly the limiting factor for phytoplankton [77,78]. The experimental results demonstrate that Fe is solubilized in the presence of this organic acid, but not by distilled water (Table 3).

### 6.1. Bioassay Methods

Bioassay methods have long been a mainstay of microbiology research, where the growth parameters of microbial cultures are used to assess the activity of various chemical agents. Many investigators have isolated microorganisms from rocks and soils, but laboratory investigations have typically involved analyses of substances produced by the organisms and their effect on geological materials. True bioassays have been performed by only a few geoscientists, e.g., [22,79]. This study shows the usefulness of bioassays for quantitatively studying the abilities of microorganisms to dissolve and metabolize nutrient elements from minerals.

For this study, two strategies were used to investigate the abilities of algae and cyanophytes to dissolve Fe from biotite and other ferruginous minerals. The isolation of pure cultures from the epilithic microbial assemblage proved to be challenging, because the gelatinous envelopes of filamentous organisms acted as carriers for other microbes. Although it was possible to obtain a pure culture of the single-celled green algae, *Palmellococcus*, attempts to obtain isolates of the other genera were unsuccessful. As discussed later, these mixed cultures were useful for study, but initially, a pure culture of a cyanophyte, *Synechococcus leopoliensis*, was used to investigate the biogenic release of Fe from biotite. The taxon chosen is a unicellular cyanophyte that grows rapidly under laboratory conditions, and which has been subject to considerable study following its original description [80], which used the now-obsolete name *Anacystis nidulans*. The microbes used in this study came from cultures maintained at Western Washington University, originally obtained from the Indiana University Culture Collection; that collection is now being maintained at the University of Texas in Austin, USA. The organism is designated *Synechococcus leopoliensis* UTEX 625 [81].

### 6.2. Bioassay of Fe Availability for Synechococcus leopoliensis Incubated with Biotite

**Figure 6:**
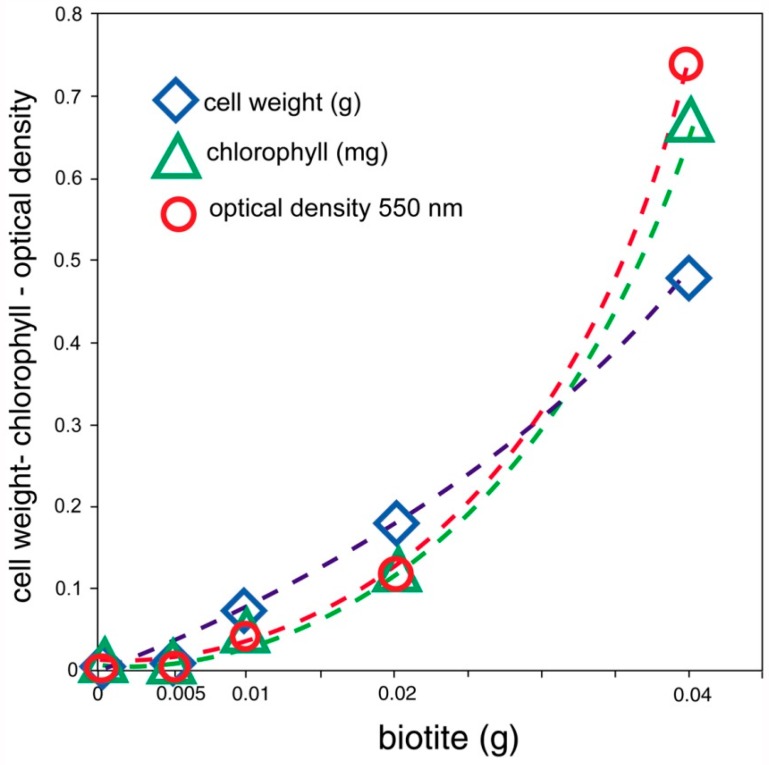
*Synechococcus leopoliensis* growth is directly related to the amount of biotite as an iron source. The exponential curve is consistent with the exponential nature of algal reproduction, which primarily results from asexual cell division.

Cultures of *S. leopoliensis* grown in iron-free culture medium show growth that is limited by the ability of the microbes to obtain this nutrient metal from an external source. For this experiment, Fe was available from varying amounts of powdered biotite. Control cultures contained either no Fe source, or water-soluble ferric citrate. The results (Table 4) show that the growth of this cyanophyte is related to its ability to obtain Fe dissolved from biotite. All three measurement parameters (cell weight, chlorophyll content, and 550 nm optical density of cell suspensions) give very similar results (Figure 6).

### 6.3. Synechococcus leopoliensis: Bioavailability of Fe from Ferruginous Minerals

**Figure 7:**
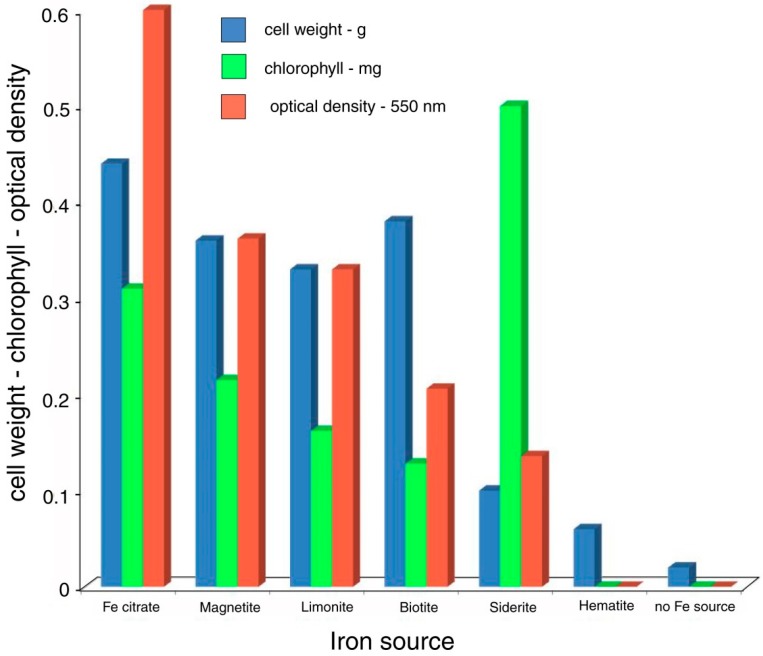
Data graph: Bioavailability of Fe from various ferruginous minerals.

The bioassay method was extended to include a variety of ferruginous minerals as potential sources of dissolved iron. As in the previous experiment, control cultures contained either no Fe source, or water-soluble ferric citrate. The results (Table 5, Figure 7) show that the availability of dissolved Fe is variable for different minerals: magnetite [Fe_3_O_4_], limonite [FeO(OH).nH_2_O], biotite [K(Mg,Fe)_3_(Al,Si)_3_O_10_)(Fe,OH)_2_], siderite [FeCO_3_], and hematite [Fe_2_O_3_]. Each mineral was present as a 0.020 g addition to the iron-free culture medium.

### 6.4. Production of Citric Acid by Epilithic Microbes Isolated from Outcrop

**Figure 8:**
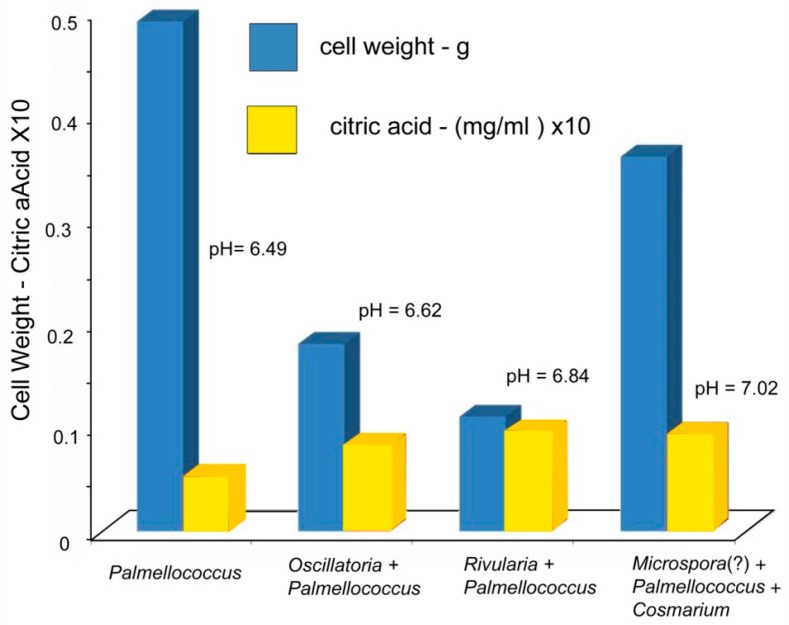
Release of citric acid by epilithic microbes.

As mentioned previously, thin-layer chromatography revealed citric acid as the only LMWOR; quantitative analysis indicated a concentration of 0.0756 mg/mL (though this concentration is probably variable according to precipitation, ambient temperature, and exposure to sunlight, all of which may affect the activity of photosynthetic organisms) Citric acid production by microbes cultured from the outcrop was measured under laboratory conditions (Table 6, Figure 8).

### 6.5. Bioassays: Mixed Cultures

The bioassay method used with the model microbe *Synechococcus leopoliensis* (Table 4) was employed to measure the ability of site-derived microbe cultures to obtain Fe from biotite, compared to control samples that contained no iron source, or water-soluble ferric citrate (Table 7).

## 7. Scanning Electron Microscopy

**Figure 9:**
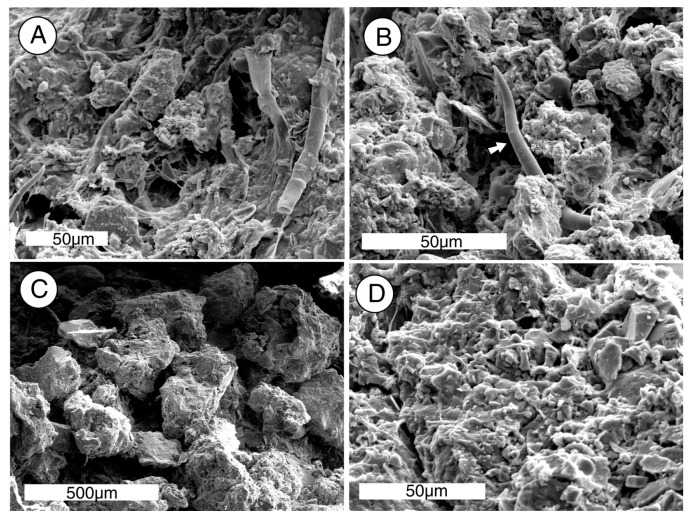
Microscopic features of Chuckanut Bay outcrop surface rock. (**A**). Sandstone is coated with microbial biofilm; (**B**). Cyanophyte trichome (marked with arrow) penetrating sand grains ~2 mm below the rock surface; (**C**). Subsurface sandstone consists of moderately well-sorted grains of silicate minerals set in a granular micaceous matrix; (**D**). High magnification view of near-surface sandstone shows an abundance of fine-grained weathering debris.

Specimens of rock collected from the outcrop surface were analyzed by SEM/EDS to observe possible evidence of biogenic weathering. The electron photomicrographs show biofilm-forming microorganisms that inhabit the surface zone of the porous arkose (Figure 9A,B), and evidence of rock weathering (Figure 9C,D).

**Figure 10:**
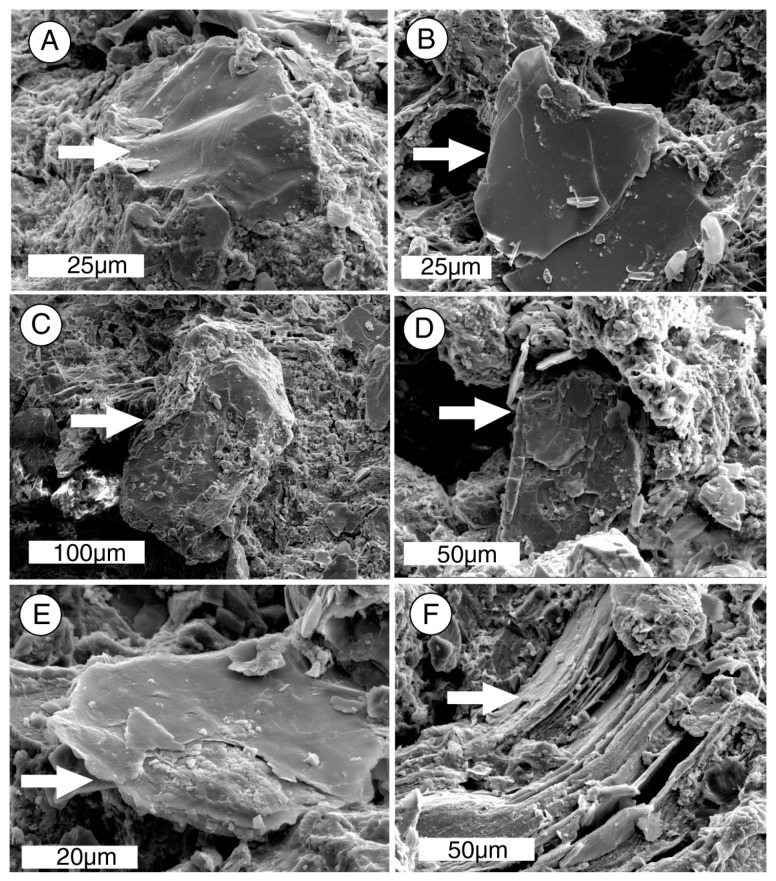
Textural characteristics of sediment grains in weathered surface zone. (**A**), quartz; (**B**), orthoclase feldspar; (**C**), hornblende; (**D**), plagioclase feldspar; (**E**,**F**), biotite mica. Biotite grains show evidence of weathering, in the form of surface etching (**E**) and delamination along platy cleavage planes (**F**). Other silicate minerals are relatively free of weathering effects.

At higher magnifications, SEM images show that most silicate grains are relatively unweathered. The exception is biotite, which shows surface pitting and delamination along the cleavage planes (Figure 10). The severity of structural damage at cleavage edges is consistent with experimental evidence that shows biotite edges are 45–120 times more susceptible to dissolution than surface planes [82].

## 8. Discussion

Field observations, microscopic observations, and the analysis of biochemicals confirm the abundant presence of photosynthetic microbes at the Chuckanut Bay site. The presence of citric acid at the moist sandstone surface suggests the possibility that the microorganisms may benefit from nutritional elements released from the mineral substrate. A laboratory experiment investigated the ability of a model organism, *Synecoccus elongates* UTEX 650, to obtain Fe from biotite and other ferruginous minerals; taxonomically mixed cultures of microbes from the rock surface were also studied. The results of these experiments are discussed below.

### 8.1. Table 4, Figure 4. Metabolism of Iron from Biotite by Synechococcus leopoliensis

These data clearly indicate that this unicellular cyanophyte is capable of dissolving Fe from biotite and metabolizing this element. Despite the rapid growth typical of this species, microbial growth did not reach a plateau point, suggesting that the dissolution of Fe occurs at a gradual rate. As a corollary, Fe does not reach a saturation point, because the element is continually being removed from solution by the expanding microbial population. This phenomenon is evidenced by data from cultures incubated with a variety of ferruginous minerals, as discussed below.

### 8.2. Table 5, Figure 5. Synechococcus leopoliensis: Bioavailability of Fe from Ferruginous Minerals

The bioavailability of Fe varies greatly among various iron-bearing minerals. Factors affecting Fe solubility may include oxidation state, lattice structure, and solubility [83]. Hematite was the only mineral that was ineffective as a nutrient source, perhaps because for this mineral, iron is present in a ferric (Fe^3+^) state, in contrast to ferrous iron (Fe^2+^) present in magnetite, biotite, and siderite. Limonite is a hydrous oxide-hydroxide where iron is present in an oxidized (Fe^3+^) state, but this mineral is anomalous because of its amorphous structure, which may facilitate solubility. The bioavailability of Fe is not limited to particular structural groups, since microbial growth occurred with oxide (magnetite), aluminosilicate (biotite), and carbonate (siderite) minerals. Bioassay results indicate that Fe in solution did not reach a saturation point, perhaps simply as a result of slow dissolution rates, but more likely a result of the removal of soluble iron complexes during microbial metabolism; under inorganic conditions, Fe saturation would eventually be reached regardless of the mineral source.

Optical density: cell weight ratios are somewhat variable among the cultures, but generally follow the same trends. The exception is the sample incubated with siderite, where chlorophyll values are very high. This phenomenon suggests that in this culture, cells contained a significantly higher chloroplast density compared to the organisms grown in the presence of other iron sources. The reason for this difference is not known, but it perhaps relates to the FeCO_3_ structure of siderite, which gives the mineral a higher solubility under acidic conditions than iron oxide and aluminosilicate minerals; siderite is susceptible to both chelation and hydronium ion attack. Why elevated Fe levels would favor increased chlorophyll production rather than simply allowing more rapid cell proliferation is unknown.

### 8.3. Table 6, Figure 6. Release of Citric Acid by Epilithic Microbes

The pH remained near neutral for all cultures, suggesting that citric acid released by microbes was being removed, presumably by the metabolism of Fe-citrate complexes. Chlorophyll level is higher in *S. leopolensis* cultures than in cultures of organisms isolated from the sandstone outcrop, which can probably be explained by the rapid growth rate typical of *Synechococcus* [68]. Citric acid levels in laboratory cultures were much lower than the concentration measured for the near-surface fluids of the sandstone outcrop. This is perhaps a result of much greater microbe population densities at the rock surface; however, the natural occurrence of microbes is subject to the influence of many physical and ecological variables not present in the simple conditions typical of laboratory cultures.

When citrate production is adjusted for cell mass, the highest citric acid levels were measured in cultures containing cyanophytes *Oscillatoria* and *Rivularia*, and lower values occurred with the green alga *Palmellococcus*. pH remained near neutral, regardless of citric acid concentration. *Palmellococcus cultures* had a relatively high cell weight, but low citric acid, a phenomenon that could be an indication of the efficient absorption of Fe-citrate chelate.

### 8.4. Table 7. Mixed Cultures & Biotite

The greatest growth was observed in the culture that contained *Rivularia* + *Palmellococcus*. Cultures that contained *Palmellococcus* alone, or in combination with *Oscillatoria*, *Microspora*, or *Cosmarium*, all had a much lower growth. This evidence suggests that *Rivularia* is the most active microbe for dissolving iron from biotite. However, under natural conditions, it is probable that Fe dissolved as a result of a chelating agent produced by one organism can be metabolized by other microbes living in the same environment. Also, metabolic processes may release elements and compounds that become available to co-inhabitants; the death of cells results in the release of useful nutrients.

### 8.5. Implications for Rock Weathering

SEM images show that the sandstone surface is covered with a continuous biofilm (Figure 9A), with microbial filaments extending several mm into the porous rock (Figure 9B). The near-surface zone shows evidence of chemical weathering. The arkosic sandstone clasts predominantly consist of quartz and feldspar, which have been relatively free of weathering damage. Hornblende, a minor constituent, is likewise well-preserved. In contrast, biotite grains show severe damage. This petrographic evidence is consistent with laboratory experiments that suggest that microorganisms are able to dissolve biotite as a source of nutrient iron. Because biotite is an important component of intergranular cement, degradation of this mineral results in the disaggregation of the sandstone.

From a regional perspective, the Chuckanut Bay study site is an anomalous coastal exposure, exposed to freshwater saturation and protected from waves, salt spray, and other marine effects because of the protection provided by the railroad causeway (Figure 2). The same Chuckanut Formation sandstone forms outcrops along a 15 km length of nearby Salish Sea coast. At other sites, outcrops contain spectacular examples of honeycomb weathering. These features result from a combination of the destructive effects of salt weathering in balance with the protective effect of microbial biofilms [69,84].

### 8.6. Possibilities for Future Research

This study provides a preliminary look at biogenic weathering processes related to mineral solubilizaton by surface-dwelling photosynthetic organisms, but many issues remain unclear. This study provides only a brief snapshot view of microbial activity during the long daylight hours and mild temperatures of the spring season; organisms may be different in other seasons. In addition, other coastal sites contain areas where the rock surface is moistened by freshwater seepage, though these sites lack the protection provided by the railroad causeway that exists at Chuckanut Bay. Weathering processes at these sites may be quite different, particularly because of the importance of salt crystallization as an agent of erosion. Evidence presented in this study provides only cursory information regarding biogenic effects, but the laboratory methods described here may be useful for future studies in other areas. In particular, the bioassay method can provide information as to how organisms may benefit from elements released via mineral dissolution.

## Figures and Tables

**Table 1:** Major element composition (wt.%) of arkosic sandstone at the Chuckanut Bay site, determined by atomic absorption spectrophotometry. Average of four analyses, data adapted from Mustoe [69].

SiO_2_	Al_2_O_3_	TiO_2_	Fe_2_O_3_ *	MgO	Na_2_O	K_2_O	CaO	MnO
72.79	13.30	0.38	3.26	2.04	3.21	1.74	1.92	0.06

* Total iron calculated as Fe_2_O_3_.

**Table 2:** Analysis of chlorophyll and lipid biomarkers in the near-surface horizon of Eocene arkosic sandstone saturated with fresh water seepage.

Sample	Chlorophyll µg/g	Total Lipids µg/g
1	9.0	156
2	10.0	244
3	5.5	266
4	7.0	128
5	7.7	228
6	5.5	129
7	8.5	44
8	20.0	442
9	6.0	140

**Table 3:** Citric acid solubilization of iron from biotite. A total of 0.5 g of powdered (<63 µm) biotite was incubated for 14 days in 50 mL of aqueous citric acid solution (0.0756 mg/mL. or in an equal volume of distilled water.

Flask	Solution	Iron Content (ppm)
1	Distilled water	0.01
2	Distilled water	<0.01
3	Citric acid	0.38
4	Citric acid	0.52

**Table 4:** Metabolism of iron from biotite by *Synechococcus leopoliensis*. Cultures were grown in 100 mL of iron-free medium for nine days under constant illumination at 25 °C in air-CO_2_ atmosphere (95:5 *v*/*v*).

Flask	Iron Source	Cell Weight/g	Chlorophyll/mg	Optical Density at 550 nm
1	Fe citrate 0.0006 g	0.53	0.74	0.668
2	Biotite 0.040 g	0.48	0.67	0.456
3	Biotite 0.020 g	0.18	0.118	0.118
4	Biotite 0.10 g	0.07	0.032	0.053
5	Biotite 0.005 g	0	0	0
6	No iron	0	0	0

**Table 5:** Bioavailability of Fe from various ferruginous minerals.

Flask	Iron Source	Cell Weight Grams	Chlorophyll mg	Optical Density at 550 nm
1	Fe citrate 0.0006 g	0.44	0.310	0.600
2	magnetite	0.36	0.215	0.362
3	limonite	0.33	0.162	0.330
4	biotite	0.38	0.128	0.206
5	siderite	0.10	0.50	0.136
6	hematite	0.06	0	0
7	No iron	0.02	0	0

**Table 6:** Release of citric acid by epilithic microbes. Cultures were grown for eight days under the same conditions as cultures used for Table 4.

Organism(s)	Cell Weight Grams	Citric Acid mg/mL	Citric Acid/Cell Weight	Final pH
*Palmellococcus*	0.49	0.00531	0.01084	6.49
*Oscillatoria* + *Palmellococcus*	0.18	0.00825	0.04583	6.62
*Rivularia* + *Palmellococcus*	0.11	0.00950	0.0864	6.84
*Microspora* + *Palmellococcus+ Cosmarium*	0.36	0.00935	0.03569	7.02

**Table 7:** Bioassay results for organisms isolated from the study site biofilm.

Organism(s) and Iron Source	Cell Weight Grams	Chlorophyll mg	Optical Density at 550 nm
*Palmellococcus*			
Fe citrate	0.46	0.057	0.322
biotite	0.57	0.048	0.404
No iron	0.29	0.012	0.157
*Oscillatoria* + *Palmellococcus*			
Fe citrate	0.40	0.015	0.178
biotite	0.39	0.015	0.080
No iron	0.26	0.003	0.002
*Rivularia* + *Palmellococcus*			
Fe citrate	0.58	0.194	0.840
biotite	0.54	0.180	0.850
No iron	0.29	0.055	0.282
*Microspora* + *Palmellococcus* + *Cosmarium*			
Fe citrate	0.91	0.055	0.432
biotite	0.75	0.038	0.352
No iron	0.28	0.006	0.008

**Figure A1:**
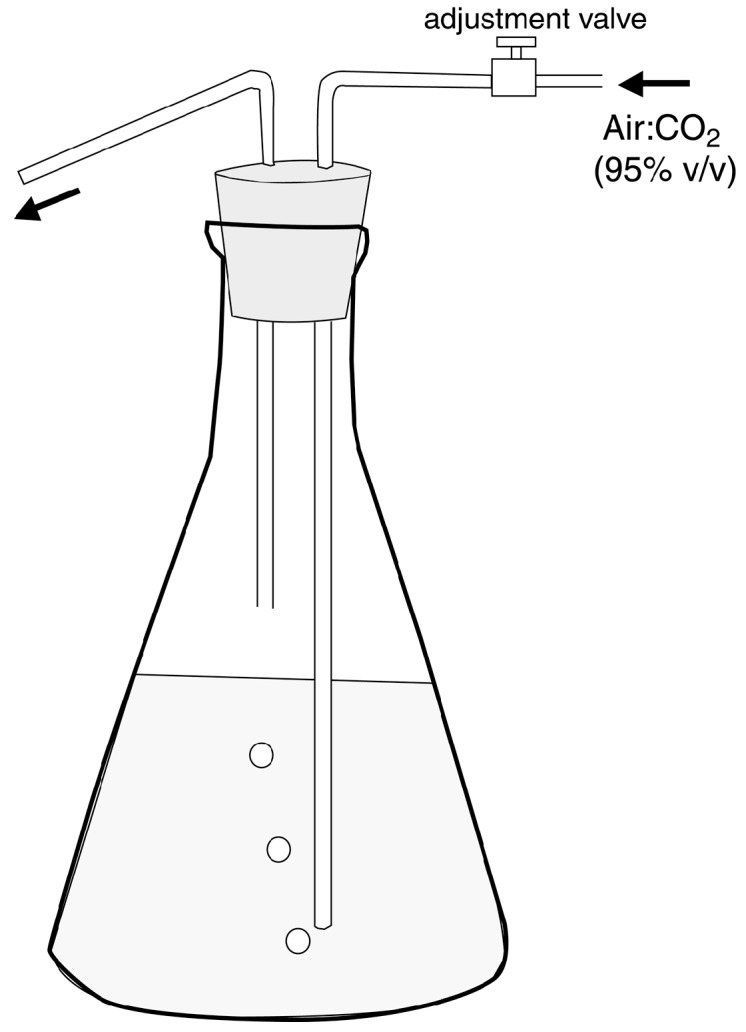
Apparatus used for microbial cultures.

## Appendix A. Methods and Materials

This study is a preliminary investigation, and the results are reported from experiments that were performed without replication. As described below, the analytical methods are based on well-documented sources, and have been used in our laboratory for many years.

### Appendix A.1. Bioassay Methods

#### Appendix A.1.1. Source of Microorganisms

*Synechococcus leopoliensis* strain 625 cultures are available from UTEX culture collection, University of Texas at Austin (https://utex.org).

#### Appendix A.1.2. Microbial Culture Medium

Adapted from Gorham et al. [85]. This culture medium contains inorganic chemicals as a source of nutrients. In the original formulation, iron is supplied in the form of ferric citrate. Because the medium consists of inorganic components, there is no likelihood of Fe being inadvertently introduced, as might happen for media prepared using organic extracts as nutrient sources. For the bioassays used in this study, ferric citrate was omitted from the recipe. For control samples, ferric citrate was prepared in aqueous solution at a concentration of 0.006 g in 100 mL distilled water. After autoclave sterilization, 10 mL of this solution was added for every 90 mL of culture medium. Prior to use, the pH of the culture medium was adjusted to 8.0 by addition of 6 N NaOH solution. The design of the individual 100 mL culture flasks is shown in Figure A1. A compressed air: CO_2_ gas mixture (95:5 *v*/*v*) was bubbled at rate of a few cm^3^/min through the culture medium. Commercial grade gas was used, custom blended for our lab by a local vendor. Our past lab experiences growing axenic algal cultures have confirmed the sterility of this product. Culture flasks were exposed to constant illumination from broad spectrum fluorescent lights. All mineral powders used in solubilization experiments were screened through a 62 µm bronze sieve.

#### Appendix A.1.3. Inoculation Procedure

For experiments using *Synechococcus*, flasks of sterile medium were inoculated by adding 1.0 mL of solution from a stock culture; the numbers of cells being transferred were not determined, but in a given experiment, each flask received the same volume of inoculum. The quantity of cells and volume of medium was low enough to minimize the amount of Fe that was transferred to flasks of iron-free medium used in bioassays.

### Appendix A.2. Analytical Methods

#### Appendix A.2.1. Cell Weight

Cell weight = weight of wet cells after centrifuging culture liquid for 7 min at 2000 g and decanting to remove supernatant.

#### Appendix A.2.2. Optical Density

Optical density is a measurement of the opacity of the culture medium caused by light absorption in solutions prepared by suspending the centrifuged cells in 400 mL distilled water, and determining the optical density at 550 nm for an aliquot transferred to the measurement cell of a Beckman Spectronic 20 spectrophotometer.

#### Appendix A.2.3. Chlorophyll Analysis

Sample collection: Rock chips were removed along a 2 m horizontal line across the outcrop using a hammer and chisel. These samples were returned to the laboratory and immediately analyzed. For each sample, 25 g of chips were pulverized by hand in a porcelain mortar, and transferred to a 125 mL Erlenmeyer flask. Chips were extracted for 60 min. in 25 mL of chloroform-methanol (2:1 *v*/*v*). The mixture was gravity filtered through medium flow filter paper (60 mL/min, Grade 613), saving the supernatant liquid. The remaining rock chips were extracted for an additional 60 min. using 25 mL of fresh solvent, and the filtered liquid was combined with the liquid from the first extraction. The absorbance of this extract was measured using a Spectronic 20 UV/VIS spectrophotometer (ThermoFisher Sciientific, Waltham, MA USA, www.thermofisher.com) at 625 µm (chlorophyll-b). For chlorophyll-a, a 675 nm wavelength can be used. Chlorophyll content was determined using a standard curve constructed by measuring the absorbance of chlorophyll extracts of a known concentration, obtained from a chloroplast suspension obtained from fresh spinach leaves [86]. For microbial cultures, chlorophyll was determined by extracting the centrifuged cells in chloroform-methanol, using two stages of extraction as described above to obtain a filtered solution that was used for spectrophotometric analysis. For both methods, during processing, samples were kept in glassware that was wrapped in aluminum foil to exclude light that could hasten chlorophyll degradation.

#### Appendix A.2.4. Lipid Analysis

The extract used for chlorophyll determination was evaporated to dryness using a rotary flash evaporator. The resulting residue was dissolved in a ~3 mL of diethyl ether, and transferred to a pre-weighed 10 mL Erlenmeyer flask. The solvent was removed using an evacuated dessicator chamber. The flask and dry reside were weighed using an analytical balance to determine the weight of total lipids.

#### Appendix A.2.5. Organic Acid Analysis

Samples consisted of rock chips collected as described above. The following analytical method was adapted from [87,88], using thin-layer chromatography instead of paper chromatography. The use of thin-layer chromatography for organic acid separations is briefly discussed in Swain [86], including a list of retardation factor (Rf) values for various carboxylic acids.

A total of 80 g of rock chips was transferred to a 250 mL Erlenmeyer flask and extracted for 60 min. using 100 mL of 95% ethanol. The mixture is filtered through Whatman #613 filter paper, saving the supernatant liquid. The sample was extracted for an additional 60 min in 50 mL of fresh solvent, then filtered. The supernatant was combined with the liquid obtained from the first extraction, and evaporated to dryness using a rotary flash evaporator. Rock chips remaining from the ethanol extraction received a second cycle of extraction, using aqueous 10% isopropanol, again employing rotary flash evaporation. Vacuum dried residues from the extraction steps were dissolved in 2 mL of the original solvent, before application on 20 × 20 × 0.4 cm glass plates coated with Silica Gel G. Carboxylic acid solutions (0.2 mg/mL, dissolved in pure ethanol or 10% aqueous isopropanol) were applied to each plate to provide reference standards. Reagent grade Krebs cycle organic acids Sigma-Aldrich, St, Louis, MO, USA, www.Sigma-Aldrich.com) included isocitric acid, citric acid, malic acid, fumaric acid, pyruvic acid, succinic acid, and oxalacetic acid.

Chromatography plates were developed in glass tanks containing 20 mL of ethanol-ammonia-water solvent (80:4:16 *v*/*v*). After development was complete (90–120 min.), the plate was removed and briefly allowed to air dry, before transfer to a 110 °C oven. Organic acids were detected using two reagents. The first step used the spray application of a pH indicator, 0.1% bromcresol green in 50% aqueous ethanol, adjusting pH to 10 with dilute aqueous NaOH. This treatment reveals organic acids as light colored spots on a blue background. The second detection step requires spraying the plate with *p*-dimethylaminobenzaldehyde in acetic anhydride (5% *v*/*v*), diluted 1:4 *v*/*v* with acetone for spray application. After heating at 110 °C, organic acids appear as pastel colored spots, which can be intensified by spraying the plate with bromcresol green indicator solution. This method provides good separation of the reference standards, with two exceptions. Oxalacetic acid was not detected by either spray reagent, while isocitric and citric acids are indistinguishable.

#### Appendix A.2.6. Quantitative Determination of Citric Acid

The procedure was adapted from Snell and Snell [89]. This colorimetric method uses a deproteinized sample with warm acetic anhydride and pyridine under anhydrous conditions, citric acid yields a carmine red color, aconitic acid yields violet red, and tartaric acid yields emerald green. Procedure: dissolve deproteinized sample containing 0.015–0.04 mg citric acid in 1 mL of 5% aqueous trichloracetic acid. Transfer sample to a glass vial, add 8 mL acetic anhydride, and seal vial with a rubber stopper. Heat in a 60 °C water bath for 10 min. Temperature should remain constant within ±1 °C. Add 1 mL pyridine, restopper, and heat vial in a water bath for an additional 40 min. Transfer to an ice bath for 5 min. Read absorbance at 420 µm using a Beckman Spectronic 20 Spectrophotometer. Consult standard curves prepared from laboratory grade citric acid to determine the concentration of unknown samples.

#### Appendix A.2.7. Determination of Dissolved Iron

Many wet chemical and instrumental methods can be used to determine values for dissolved iron. If available, atomic absorption spectrophotometery and ICP spectrophotometery are common methods. Colorimeteric analysis was used in this study, using 1, 10-phenanthroline (orthophenanthroline) to produce a bright colored Fe(II) complex. The method is rapid, requires minimal laboratory facilities, and has detection sensitivities in the <1 ppm range [89,90,91].

Procedure: 25 mL of the liquid sample is mixed with 0.25 mL of 10% aqueous hydroxlamine hydrochloride to convert Fe^3+^ to Fe^3+^. Add 5 mL of 0.1% aqueous 1,10-phenanthroline. Absorbance is determined at 508 µm using a Spectronic 20 spectrophotometer, calculating Fe concentrations based on a standard curve prepared from solutions with a known iron concentration, prepared using reagent grade ferric citrate dissolved in distilled H_2_O.

#### Appendix A.2.8. Scanning Electron Microscopy

Photomicrographs (Figure 9 and Figure 10) were obtained using a Tescan VEGA SEM (Tescan, Brno, Czech Republic, www:tescan.com) at Western Washington University. Specimens were prepared as rock chips attached to aluminum stubs with epoxy adhesive, and sputter coated with palladium to provide electrical conductivity. Samples were dried at 110 °C prior to analysis. Mineral phases were identified based on EDS spectra, using an EDAX nondispersive X-ray detector running Genesis software (EDAX, Mahwah, NJ, USA, www:edax.com). Scale bars were added using Adobe Illustrator.
